# Experimental Study of Informal Rewards in Peer Production

**DOI:** 10.1371/journal.pone.0034358

**Published:** 2012-03-29

**Authors:** Michael Restivo, Arnout van de Rijt

**Affiliations:** Department of Sociology, State University of New York at Stony Brook, New York, New York, United States of America; University of Maribor, Slovenia

## Abstract

We test the effects of informal rewards in online peer production. Using a randomized, experimental design, we assigned editing awards or “barnstars” to a subset of the 1% most productive Wikipedia contributors. Comparison with the control group shows that receiving a barnstar increases productivity by 60% and makes contributors six times more likely to receive additional barnstars from other community members, revealing that informal rewards significantly impact individual effort.

## Introduction

Internet-enabled peer production makes it possible for organizations to pool work from geographically-dispersed volunteers to create non-trivial goods and services [1], [2]. One striking example is the online encyclopedia Wikipedia, where production of content follows a long-tailed distribution carried by a core of extremely active individuals [3], while consumption is dispersed across a vast population of free-riders [4], [5]. This raises the question of what accounts for time-intensive contributions to large-scale peer production efforts by socially-unembedded and oftentimes anonymous individuals in the absence of salary or contract [6].

In peer production, it is believed that informal rewards from fellow contributors substitute for material incentives to dedicate time and resources. Wikipedia contributors can award their peers a barnstar – an editing award – by posting it on a user's page for public display. Receiving a barnstar indicates that one's efforts are recognized as valuable and is thought to act as a sign of prestige within the community [7]. A survey of Wikipedia contributors by the Wikimedia Foundation concluded that “positive interactions like receiving compliments and barnstars from fellow editors … made them more likely to edit Wikipedia” [8]. This idea is consistent with social scientific theories suggesting that positive incentives such as rewards, social recognition, peer esteem, and accrual of status can serve as motivations for contributing to public goods more generally [9]–[13].

Existing approaches to evaluating the effects of informal rewards in peer production have been inconclusive. First, this is because contributors' self-reports on how rewards affect them are of questionable reliability due to evaluation or perceptual biases. Second, in longitudinal records of contributor behavior, contribution and reward histories co-evolve making it difficult to disentangle cause from effect. To overcome these challenges, we conducted a controlled experiment under naturalistic conditions in which informal rewards were randomly allocated to a real-world population of Wikipedia contributors.

## Methods

This study's research protocol was approved by the Committees on Research Involving Human Subjects (IRB) at the State University of New York at Stony Brook (CORIHS #2011-1394). Because the experiment presented only minimal risks to subjects, the IRB committee determined that obtaining prior informed consent from participants was not required. Confidentiality of personally-identifiable information has been maintained in strict accordance with Human Subjects Committee requirements for privacy safeguards.

We designed our experiment to test the hypothesis that informal rewards have a reinforcing effect on volunteer work effort. We performed our experiment on a random sample of 200 active contributors from among the 1% most productive editors who had never been awarded a barnstar from another user. To construct our sampling frame, we obtained a list of active Wikipedia contributors – defined as any user who performed at least 1 edit (modification to the English Wikipedia project) in the 30-day window prior to the start of the experiment. We ranked this population of 144,120 contributors by their total number of edits, after which we screened into our sampling frame the top 1% of users and discarded the remaining. Next, we eliminated any high-volume contributors who had previously received a barnstar or had elevated administrative privileges in the community. We then took a uniformly random sample of 200 users and through random assignment either awarded a barnstar (100 cases) or withheld the award in the control group (100 cases). Finally, we observed all 200 subjects' actions for 90 days.

After the observation period ended, we compared contributors' productivity (article modifications) and peer recognition (additional barnstars received from other users) across conditions. To account for between-subject differences in pre-treatment productivity, we calculated cumulative productivity on any day as the running total number of article modifications divided by the number of article modifications in the 30-day pre-treatment period. To test the null hypothesis that post-treatment productivity would be equal across conditions, we employed a measure of central tendency (median) and a non-parametric test (Mann-Whitney U test) that are robust to outliers and distributional skew. To test for an experimental effect in subsequent peer recognition, we performed a Pearson chi-square test (χ^2^).

## Results and Discussion

In both groups, median productivity was lower after the treatment, which can be attributed to regression toward the mean – resulting from our sampling the 1% most productive users – as well as to general turnover in the contributor population. However, users who received a barnstar exhibited greater sustained productivity and were less likely to discontinue contributing. Of 19 users who made zero edits in the post-experiment observation period, only five received the experimental treatment (χ^2^ = 4. 711, df = 1, p = 0.030).

**Figure 1:**
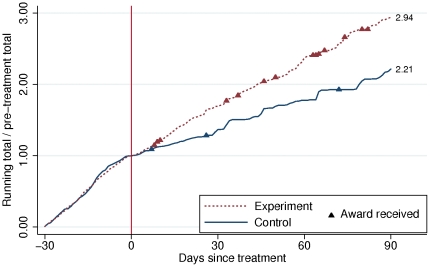
Median cumulative productivity by experimental condition. Cumulative productivity is measured as the running total number of post-treatment edits to encyclopedic articles divided by the total number of such edits made during the 30 days prior to the treatment for each subject. The treatment was given on day 0 (vertical line). By the end of the 90-day observation period, subjects in the experimental group exhibited a median productivity that was 2.94 times their pre-treatment total versus 2.21 in the control group, for a post-treatment difference of 60%. Also shown are additional awards received by subjects from third-parties after the treatment. Twelve subjects in the experimental condition received a total of fourteen awards, whereas two subjects in the control condition received a total of three awards.

We also find significant between-group differences in productivity: receiving a barnstar increased median productivity by 60% compared to the control group (Mann-Whitney U-test: z = 3.222; p = 0.001), shown in Figure 1. Other tests (median test, Student's T test) also yield significant differences between conditions (p<0.01). The magnitude of this difference remained approximately constant over the course of the 90-day observation period, suggesting that the barnstars we awarded had a sustained effect on productivity. In addition to exhibiting greater productivity, subjects in the experimental condition were significantly more likely to receive additional rewards from other contributors. Twelve experimental subjects were subsequently awarded one or more barnstars from other contributors, compared to two subjects in the control group (χ^2^ = 7.681, df = 1, p = 0.006). These twelve individuals exhibited no greater productivity prior to receiving the additional barnstars when compared to others in the experimental condition (Mann-Whitney U-test: z = .743; p = 0.458). For this test, productivity was calculated as the total number of edits up to day 8, when the first additional barnstar was awarded. The result of the test remains unchanged when productivity is calculated up to day 82, when the last additional barnstar was awarded (Mann-Whitney U-test: z = .796; p = 0.426). This suggests that cumulative advantage [14], [15] in the allocation of informal rewards operates through a mechanism of enhanced social prestige in the community and not increased merit.

The findings demonstrate that even though informal rewards are free to give and carry no immediate material benefits, they have a substantial positive effect on the productivity of Wikipedia contributors. Our findings indicate that this beneficial effect can become self-reinforcing as reward-receiving accumulates for recipients. Together, these results suggest that the facilitating role of informal rewards in peer production systems derives from their ability to stimulate individual effort as well as contribute to an accrual of social recognition. While previous scholars suggest that the intensity of informal rewards in peer production is low [6], the present research quantifies their magnitude and indicates that informal rewards may play a key role in sustaining volunteer effort.
